# Anti-inflammatory effects of royal jelly on ethylene glycol induced renal inflammation in rats

**DOI:** 10.1590/S1677-5538.IBJU.2014.0470

**Published:** 2015

**Authors:** Zeyneb Aslan, Laçine Aksoy

**Affiliations:** 1 Department of Chemistry, Biochemistry Division, Faculty of Science and Arts, Afyon Kocatepe University, Afyonkarahisar, Turkey

**Keywords:** Calcium Oxalate, Inflammation, royal jelly [Supplementary Concept], Ethylene Glycol, Cytokines

## Abstract

**Objective:**

In this study, anti-inflammatory effects of Royal Jelly were investigated by inducing renal inflammation in rats with the use of ethylene glycol. For this purpose, the calcium oxalate urolithiasis model was obtained by feeding rats with ethylene glycol in drinking water.

**Materials and Methods:**

The rats were divided in five study groups. The 1^st^ group was determined as the control group. The rats in the 2^nd^ group received ethylene glycol (1%) in drinking water. The rats in the 3^rd^ group were daily fed with Royal Jelly by using oral gavage. The 4^th^ group was determined as the preventive group and the rats were fed with ethylene glycol (1%) in drinking water while receiving Royal Jelly via oral gavage. The 5^th^ group was determined as the therapeutic group and received ethylene glycol in drinking water during the first 2 weeks of the study and Royal Jelly via oral gavage during the last 2 weeks of the study.

**Results:**

At the end of the study, proinflammatory/anti-inflammatory cytokines, TNF-α, IL-1β and IL-18 levels in blood and renal tissue samples from the rats used in the application were measured.

**Conclusion:**

The results have shown that ethylene glycol does induce inflammation and renal damage. This can cause the formation of reactive oxygen species. Royal Jelly is also considered to have anti-inflammatory effects due to its possible antiradical and antioxidative effects. It can have positive effects on both the prevention of urolithiasis and possible inflammation during the existing urolithiasis and support the medical treatment.

## INTRODUCTION

Urinary system stone disease is a frequent disease that can adversely affect the quality of life. Various factors such as genetic factors, characteristics of the residential area and their effects on the metabolism, dietary habits, smoking, alcohol use, air pollution and stress are considered to trigger stone formation. In the urinary system stone disease, genetic factors have a 25% effect on individuals. Urinary system stone disease is three times more common in men than in women. Although it can be seen at any age, its frequency increases with age and it is most commonly seen in individuals between the ages of 30 and 60 (1–4). India and European and Middle Eastern countries are determined as the stone zone and Turkey is one of these countries in which the urinary system stone disease is endemic (5). Fluid intake has a significant effect on stone formation. Leading to increased concentration in the urine content, urine volume decreasing with decreased water consumption affects stone formation. Increased water consumption accelerates the urinary system cycle and increases urine volume and therefore, reduces stone formation (6).

ESWL (Extracorporeal shock wave lithotripsy) is the most frequently used method for the prevention of stone formation and the growth of the existing stone and litholysis, if applicable. The reliable technology and therapeutic effect of ESWL rendered it the most preferred method. However, the success of the therapy varies with the location and structure of the stone and the presence of anatomical anomalies. Despite yielding high disintegration levels, the ESWL method carries some risks. In the long term, increase in systemic blood pressure, decrease in the renal function and increase in the recurrence of calcium oxalate urolithiasis occur. Some experimental studies determined that the ESWL method causes acute and chronic damages to kidneys and some of these damages are irreversible. Patient selection is crucial to the success of the ESWL method. Applications on pregnant women and short children and applications with the presence of contraindications to anesthesia, untreated urinary system infections, active tuberculosis infection, stenosis of the urinary tract, obesity or skeletal abnormalities are difficult (6, 7).

Due to its attributed extraordinary biological properties, Royal Jelly is a significant commercial attraction for the health researches and the food industry. It is used in a wide variety of industries such as the drug, food and cosmetic industries. Royal jelly is a substance secreted from the hypopharyngeal and mandibular glands of 5-15 days old worker honey bees. It is produced in the glands of adult worker bees fed with honey and pollen (8–10).

The aim of this study was to obtain ethylene glycol induced urolithiasis in rats and to investigate the proinflammatory and anti-inflammatory effects of Royal Jelly on ethylene glycol induced urolithiasis and inflammation due to renal tubular damage by measuring cytokine levels.

## MATERIALS AND METHODS

### Chemicals

Ethylene glycol used to induce urolithiasis was obtained from Merck (Darmstadt, Germany). Royal Jelly was directly supplied from a natural honey producer (Kahramanmaraş, Turkey).

### Stone-forming rat model and experimental design

Before the initiation of the research, ethics committee approval dated 26/09/2013 no. AKÜHADYEK-284-13 was obtained from Afyon Kocatepe University Animal Ethics Committee. 35 male Sprague-Dawley strain rats weighing 300380 g were used in the study. For 4 weeks, rats were kept in 12 hours ideal lighting and at the appropriate temperature and fed with standard pellet rat feed (Bilyem, Ankara, Turkey). Rats were divided into 5 groups. Group 1, Control group (n=7): Fed with standard feed and drinking water. Group 2: EG Group (n=7): Fed with standard feed and drinking water containing 1% ethylene glycol. Group 3: RJ group (n=7): Daily fed with 100mg/kg Royal Jelly by using oral gavage. Group 4: RJ+EG group (n=7): Received 1% ethylene glycol containing drinking water. Additionally, these rats were daily fed with 100mg/kg Royal Jelly via oral gavage. Group 5: EG+RJ group (n=7): Received 1% ethylene glycol containing drinking water for the first 2 weeks. In the last 2 weeks, rats were fed with 100mg/kg Royal Jelly via oral gavage. EG represents the urolithiasis group; RJ+EG represents the prophylactic group; EG+RJ represents the therapeutic group.

### Evaluation of Total Protein Levels

The total protein levels of tissue homogenates were measured with ELISA (BIOTEK ELx800) using commercial kits (Fluka 51254). The Bradford method was used in the measurement. Coomassie Brillant Blue G-250, the dye used in this method, has a negative charge and binds to the positive charges on the surface of protein. The dye has blue and red forms. Protein binding turns the blue form into the red form. The absorbance of the resulting complex was measured at 630nm (11). Total protein results were used to calculate the levels of the parameters analyzed in kidney tissue.

### Evaluation of TNF-α, IL-1β, IL-18 Cytokine Levels

Inflammation markers (TNF-α, IL-1β,IL-18) were measured in renal homogenates and plasma samples by using commercial kits. The kits used in the measurement have the following brand, reference and lot numbers: TNF-α (eBioscience, Ref: BMS622, Lot: 91475038), IL-1β (eBioscience, Ref: BMS630, Lot: 87225015), IL-18 (Novex, Ref: KRC2341, Lot: 130401/A). Cytokine levels were given as pg/mL for plasma and pg/ mg-protein for renal homogenates.

### Statistical analysis

The data were expressed as the mean±standard deviation (SD). Statistical comparisons were performed using the appropriate ANOVA model with Duncan post-hoc tests. Differences with p<0.05 were considered significant. The SPSS 15.0 package for Windows was used for the statistical analysis.

## RESULTS

### Plasma and renal tissue TNF-α Levels

Based on the plasma TNF-α values shown in Table-1, the value of the control group did not significantly differ from the value of the Royal Jelly group (p>0.05), whereas it differed significantly from the other groups (p<0.05). The TNF-α values of the EG and EG+RJ groups were signifi-cantly the highest (p<0.05). The TNF-α value of the RJ+EG group was significantly lower than those of the EG and EG+RJ groups (p<0.05), whereas it was significantly higher than the values of the control and the RJ groups (p<0.05).

**Table 1:** Plasma and renal tissue TNF-α Levels.

Group	Plasma TNF-α (pg/mL)	Kidney TNF-α (pg/mg-protein)
Control	43.78±3.81^b^	413.26±17.78^c^
EG	75.54±9.34^a^	469.28±28.96^a^
RJ	49.96±2.65^b^	343.16±24.28^b^
RJ+ EG	59.07±5.79^c^	361.68±15.18^b^
EG+RJ	81.27±5.24^a^	474.14±15.87^a^

**a, b, c, d:** Differences between the groups that are coded with different letters in the same column are significant (p<0.05). EG represents the group administered with ethylene glycol; Royal Jelly represents the group solely administered with Royal Jelly; RJ+EG represents the group simultaneously administered with Royal Jelly and ethylene glycol; EG+RJ represents the group administered with Royal Jelly after inducing urolithiasis with ethylene glycol.

### Plasma and renal tissue IL-1β Levels

Based on the plasma IL-1β values shown in Table-2, the value of the control group was significantly lower than the other groups (p<0.05). The IL-1β value of the EG group was significantly the highest (p<0.05). The IL-1β value of the RJ group was significantly higher than the values of the RJ+EG and EG+RJ groups (p<0.05).

**Table 2:** Plasma and renal tissue IL-1β Levels.

Group	Plazma IL-1β (pg/mL)	Kidney IL-1β (pg/mg-protein)
Control	99.17±2.79^b^	1252.19±90.42^b^
EG	165.33±10.25^a^	2162.49±54.93^a^
RJ	113.17±5.12^c^	1559.00±99.30^d^
RJ+ EG	146.67±10.42^d^	1488.46±117.48^d^
EG+RJ	138.00±7.72^d^	1361.47±73.01^c^

**a, b, c, d:** Differences between the groups that are coded with different letters in the same column are significant (p<0.05). EG represents the group administered with ethylene glycol; Royal Jelly represents the group solely administered with Royal Jelly; RJ+EG represents the group simultaneously administered with Royal Jelly and ethylene glycol; EG+RJ represents the group administered with Royal Jelly after inducing urolithiasis with ethylene glycol.

Based on the renal IL-1β values; the value of the control group was significantly lower than the other groups (p<0.05). The difference between the values of the RJ group and RJ+EG groups were not significant (p>0.05), whereas their values were significantly lower than those of the control and EG+RJ groups (p<0.05). The IL-1β value of the EG group was significantly the highest (p<0.05) (Table-2).

### Plasma and renal tissue IL-18 Levels

Based on the plasma IL-18 values shown in Table-3, the IL-18 value of the control group was significantly the lowest (p<0.05). The values of the RJ group and EG+RJ groups were not significantly different from each other (p<0.05), whereas their values were significantly lower than the value of the RJ+EG group (p<0.05). The IL-18 value of the EG group showed a significantly higher increase (p<0.05).

**Table 3:** Plasma and renal tissue IL-18 Levels.

Group	Plasma IL-18 (pg/mL)	Kidney IL-18 (pg/mg-protein)
Control	2285.30±103.57^b^	980.02±42.16^b^
EG	3577.76±135.95^a^	1258.35±89.96^a^
RJ	2572.73±121.82^c^	1050.45±86.33^b^
RJ+ EG	3111.12±71.80^d^	1063.43±92.30^b^
EG+RJ	2683.17±109.96^c^	1037.43±75.48^b^

**a, b, c, d:** Differences between the groups that are coded with different letters in the same column are significant (p<0.05). EG represents the group administered with ethylene glycol; Royal Jelly represents the group solely administered with Royal Jelly; RJ+EG represents the group simultaneously administered with Royal Jelly and ethylene glycol; EG+RJ represents the group administered with Royal Jelly after inducing urolithiasis with ethylene glycol.

The analysis of the renal IL-18 values showed that only IL-18 value of the EG group was significantly higher than the other groups (p<0.05) (Table-3).

## DISCUSSION

Inflammation is a natural defense response of the organism against stimuli, such as trauma, infectious agents, toxic and chemical substances, physical and surgical operations. Ethylene glycol is a highly preferred agent to induce nephropathy in experimental animal studies. The administration of ethylene glycol results in intrarenal CaO_x_ deposition and acute renal damage. Various studies have shown that CaO_x_ deposition causes renal inflammation (12).

Proinflammatory cytokines are released at the onset of the inflammation and they increase the release of other cytokines and activate inflammatory cells. They are essential to the initiation and sustenance of immune response. Main proinflammatory cytokines in human immune response are TNF-α, IL–1 and IL-18 (13). TNF-α is a cytokine produced mainly by monocytes/macrophages, although it can be produced by Kuppfer cells, skin keratinocytes, brain glial cells, T lymphocytes and B lymphocytes. Various tissues such as liver, muscle, intestinal and lung tissues have high-affinity receptors for TNF-α. It is primarily released and also the strongest among the proinflammatory cytokines as a mediator in host response. It stimulates mononuclear phagocytes and other cells to produce IL-1, IL-6 and chemokines (14, 15). IL-1ß is a proinflammatory cytokine that has effects similar to those of TNF-α. TNF-α increases the synthesis and release of IL-1α and IL-1ß which are released from macrophages and endothelial cells and coded by different genes. IL-1ß is responsible for the metabolic and physiological effects of circulatory TNF-α. It has a role in the local and systemic effects of acute and chronic inflammation (16). IL-18 is an IFN-γ producing proinflammatory cytokine. IL-18 is released from renal tubular cells and macrophages. It functions as a mediator in acute renal damage. Studies have shown that IL-18 levels increase in the proximal tubular epithelium in the case of renal damage and acute renal failure (17).

Apitherapy means the use of bee products for medical purposes. In the recent years, there is a growing interest, especially in the food industry, in the functional foods which are claimed to have benefits for human health. The benefits of consumed food on human health are globally accepted. Today, widely known bee products, honey, propolis and Royal Jelly have a significant place among the functional foods (18).

Many studies on the effects of apitherapeutical products on inflammation induced by various causes were conducted. Khayyal et al. (19) considered propolis as an anti-inflammatory agent due to its inhibition of platelet aggregation and eicosanoid synthesis and investigated its effects on edema and arthritis induced inflammation. The researchers have found that it shows anti-inflammatory effects depending on the dosage. Another study to investigate the anti-inflammatory effects of honey on inflammation due to intestinal diseases showed that application of honey is as effective as treatment with prednisolone. However, it was stated that further studies are required to clarify the mechanism (20). In another study, paw edema was induced in rats and the effects of ethanolic extract of pollen were investigated. It was reported that pollen shows anti-inflammatory effects by reducing NO production and COX-2 activity (21). Similar to those studies, the TNF-α values in our study (Table-1) showed that the administration of EG increases TNF-α values in plasma and kidneys. In plasma and kidney, the TNF-α values of the RJ+EG group showed that Royal Jelly has preventive effects on inflammation.

In the study to investigate effects of propolis on stress-induced immunosuppression, IL-1β and IL-6 cytokine levels were examined. The researchers reported that propolis cannot substitute for inhibitory effects on proinflammatory cytokines but partially regulates TLR2 mRNA expression and balances inhibition of TLR-4 expression in stressed rats (22).

The IL-1β values in our study showed that the urolithiasis group had an increased release of the cytokine when compared to the control group. When the RJ+EG and EG+RJ groups are compared with the urolithiasis group, it is possible to say that Royal Jelly has anti-inflammatory effects. In renal tissue, the anti-inflammatory effect of Royal Jelly is higher in the therapeutic group. Renal IL-1β values show that Royal Jelly has an effect on EG-induced inflammation (Table-2).

Parikh et al. (23) published a study about cytokine IL-18 as an early diagnostic biomarker of acute renal damage. In their study on mice, Faubel et al. (24) determined that cisplatin induced renal damage is associated with the increase in IL-1β and IL-18 cytokines.

The IL-18 values in our study clearly showed the effect of exposure to ethylene glycol on cytokine release. In the plasma, the effect of Royal Jelly was higher on the therapeutic group than the control group. Data on renal tissue show that the release of IL-18 in this tissue is remarkably inhibited by Royal Jelly. Due to the insignificant differences from the control group, the values of the Royal Jelly, RJ+EG and EG+RJ groups support this assessment (Table-3).

## CONCLUSIONS

Various studies showed that oxidative stress occurs in patients with kidney stones. CaO_x_ exposure causes oxidative damage by reactive oxygen species such as superoxide and H_2_O_2_. The produced ROS activate several signaling pathways.

This study has shown that inflammation can be induced by ethylene glycol. The favorable effects of Royal Jelly on renal damage due to inflammation are presented. The resulting renal damage is associated with oxidative stress. Antioxidants in Royal Jelly prevent ROS production and support the antioxidant system. Hence, Royal Jelly is considered to show anti-inflammatory effects by affecting the signaling pathways. Further cell culture studies will help to clarify the mechanism.

## References

[1] Sowers MR, Jannausch M, Wood C, Pope SK, Lachance LL, Peterson B (1998). Prevalence of renal stones in a population-based study with dietary calcium, oxalate, and medication exposures. Am J Epidemiol.

[2] Evan AP (2010). Physiopathology and etiology of stone formation in the kidney and the urinary tract. Pediatr Nephrol.

[3] Lerolle N, Lantz B, Paillard F, Gattegno B, Flahault A, Ronco P (2002). Risk factors for nephrolithiasis in patients with familial idiopathic hypercalciuria. Am J Med.

[4] Favus MJ (1989). Familial forms of hypercalciuria. J Urol.

[5] Erkurt B, Caskurlu T, Atis G, Gurbuz C, Arikan O, Pelit ES (2014). Treatment of renal stones with flexible ureteroscopy in preschool age children. Urolithiasis.

[6] Abbagani S, Gundimeda SD, Varre S, Ponnala D, Mundluru HP (2010). Kidney stone disease: etiology and evaluation. Int J Appl Biol Pharm.

[7] Younesi Rostami M, Taghipour-Gorgikolai M, Sharifian R (2012). Treatment of Kidney Stones Using Extracorporeal Shock Wave Lithotripsy (ESWL) and Double-J Stent in Infants. Adv Urol.

[8] Sabatini AG, Marcazzan GL, Caboni MF, Bogdanov S, Almeida-Muradian LB (2009). Quality and standardisation of Royal Jelly. JAAS.

[9] Lercker G, Capella P, Conte LS, Ruini F, Giordiani G (1981). Components of Royal Jelly: Identification of the organic acids. Lipids.

[10] Brouwers EM, Ebert R, Beetsma J (1987). Behavioural and physiological aspects of nurse bees in relation to the composition of larval food during caste differentiation in the honeybee. J Apic Res.

[11] Bradford MM (1976). A rapid and sensitive method for the quantitation of microgram quantities of protein utilizing the principle of protein-dye binding. Anal Biochem.

[12] Khan SR (2005). Hyperoxaluria-induced oxidative stress and antioxidants for renal protection. Urol Res.

[13] Akcay A, Nguyen Q, Edelstein CL (2009). Mediators of inflammation in acute kidney injury. Mediators Inflamm.

[14] Hamdi H, Mariette X, Godot V, Weldingh K, Hamid AM, Prejean MV (2006). Inhibition of anti-tuberculosis T-lymphocyte function with tumour necrosis factor antagonists. Arthritis Res Ther.

[15] Powell CB, Scott JH, Collins JL (1998). Comparison of TNFalpha and TNFbeta cytolytic mechanisms in human ovarian and cervical carcinoma cell lines. Gynecol Oncol.

[16] Guo CJ, Douglas SD, Lai JP, Pleasure DE, Li Y, Williams M (2003). Interleukin-1beta stimulates macrophage inflammatory protein-1alpha and-1beta expression in human neuronal cells (NT2-N). J Neurochem.

[17] Schindler H, Lutz MB, Röllinghoff M, Bogdan C (2001). The production of IFN-gamma by IL-12/IL-18-activated macrophages requires STAT4 signaling and is inhibited by IL-4. J Immunol.

[18] Ramadan MF, Al-Ghamdi A (2012). Bioactive compounds and health-promoting properties of royal jelly: A review. J Funct Foods.

[19] Khayyal MT, el-Ghazaly MA, el-Khatib AS (1993). Mechanisms involved in the antiinflammatory effect of propolis extract. Drugs Exp Clin Res.

[20] Bilsel Y, Bugra D, Yamaner S, Bulut T, Cevikbas U, Turkoglu U (2002). Could honey have a place in colitis therapy? Effects of honey, prednisolone, and disulfiram on inflammation, nitric oxide, and free radical formation. Dig Surg.

[21] Maruyama H, Sakamoto T, Araki Y, Hara H (2010). Anti-inflammatory effect of bee pollen ethanol extract from Cistus sp. of Spanish on carrageenan-induced rat hind paw edema. BMC Complement Altern Med.

[22] Pagliarone AC, Orsatti CL, Búfalo MC, Missima F, Bachiega TF, Júnior JP (2009). Propolis effects on pro-inflammatory cytokine production and Toll-like receptor 2 and 4 expression in stressed mice. Int Immunopharmacol.

[23] Parikh CR, Mishra J, Thiessen-Philbrook H, Dursun B, Ma Q, Kelly C (2006). Urinary IL-18 is an early predictive biomarker of acute kidney injury after cardiac surgery. Kidney Int.

[24] Faubel S, Lewis EC, Reznikov L, Ljubanovic D, Hoke TS, Somerset H (2007). Cisplatin-induced acute renal failure is associated with an increase in the cytokines interleukin (IL)1beta, IL-18, IL-6, and neutrophil infiltration in the kidney. J Pharmacol Exp Ther.

